# A New Lining for Vulcanite Plates

**Published:** 1893-04

**Authors:** L. Crowther

**Affiliations:** Laurel, Md.


ARTICLE VIII.

### A NEW LINING FOR VULCANITE PLATES.

BY L. CROWTHER, D. D S., LAUREL, MD.

Rubber dam as a lining for vulcanite plates cannot be surpassed. You proceed as usual with your case and when ready for packing, first pack round the pins and flange; then cut a piece of red rubber the shape and size of your cast, large enough to come up as high as you will require your case when finished. Then lay a new cream thin piece of rubber dam on this and cut out a piece to fit, remove and paint your red plate all over with good red or black rubber solder or cement on one side, being careful that it is all covered well with the cement. Now take the piece of dam and place it smoothly on the painted side of your plate; press well down; make it quite smooth, being sure there are no air bubbles. If your dam has stretched, which it will, trim the edges to the red plate. Place your plate in the flask so that your dam will come next your cast. When you close your flask, be sure and see that the plate comes well up round the flange so as to hug close to the model and not allow any red rubber to be forced inside. Close your case by dry heat. Use paper vacums, and not tin as the dam will not harden over tin.

Rubber dam is better than gold for a lining as it is an non-conductor, prevents sore mouths and makes a very tough plate almost impossible to break; so you can make a thin, light piece of work.

---

